# Comparison of Volatile Constituents Present in Commercial and Lab-Distilled Frankincense (*Boswellia carteri*) Essential Oils for Authentication

**DOI:** 10.3390/plants11162134

**Published:** 2022-08-16

**Authors:** Pawan Kumar Ojha, Darbin Kumar Poudel, Anil Rokaya, Rakesh Satyal, William N. Setzer, Prabodh Satyal

**Affiliations:** 1 Analytica Research Center, Kritipur, Kathmandu 446088, Nepal; 2 Aromatic Plant Research Center, 230 N 1200 E Suite 100, Lehi, UT 84043, USA; 3 Department of Chemistry, University of Alabama in Huntsville, Huntsville, AL 35899, USA

**Keywords:** adulteration, enantiomeric distributions, commercial oils, α-pinene, biomarker

## Abstract

A comparative analysis of the chemical constituents present in twenty-one commercial and two lab-distilled frankincense (*Boswellia carteri*) essential oils was carried out using gas chromatography-mass spectrometry (GC-MS) and chiral gas chromatography-mass spectrometry (CGC-MS) for authentication. Out of the twenty-one commercial samples, six were adulterated with synthetic limonene, three were contaminated with synthetic octyl acetate, three were adulterated with castor oil, and two samples each were contaminated with frankincense resin and *Boswellia occulta* species, respectively, and one was contaminated with the *Boswellia serrata* species. Additionally, one sample was contaminated with phthalates as well as a cheap essential oil with similar compositions. Furthermore, one sample was adulterated with copaiba resin and frankincense resin in combination with synthetic octyl acetate. Additionally, one was contaminated with *Boswellia serrata* species, which was further adulterated with castor oil and frankincense resin. To the best of our knowledge, this is the first report to compare the enantiomeric distribution of chiral terpenoids present in commercial frankincense essential oil with lab-distilled frankincense oil for authentication. The CGC-MS analysis showed the presence of a total of eight chiral terpenoids in lab-distilled frankincense essential oils, which can be used as chemical fingerprints for the authentication of frankincense essential oil.

## 1. Introduction

Frankincense oil, commonly known as the king of essential oil, is extracted by the hydro-distillation or steam distillation of aromatic oleo-gum resin produced by the trees of the *Boswellia* genus. There are around twenty species of *Boswellia* distributed across Africa, Arabia, and South Asia. The oleo-gum resin has been used in traditional medicine, cultural, and religious ceremonies since ancient times, but now, the essential oil derived from these resins has become popular in perfumery and aromatherapy [1]. Among the various species of *Boswellia*, only a few are traded in a significant amount: *B. sacra, B. carteri, B. frereana, B. papyrifera,* and *B. serrata*. The highest quality frankincense resins are produced by *B. carteri* trees [2].

The essential oil of frankincense can be distinguished based on its chemical composition. However, various factors such as climate, geographical location, and harvesting period may also affect the chemical compositions of frankincense essential oils [3]. *B. carteri* resins, native to Somaliland and Somalia, are rich in α-pinene and limonene while Omani *B. sacra* species are dominated by α-pinene [4,5]. Similarly, *B. frereana* grown in northern Somalia are rich in α-pinene, α-thujene, and *p*-cymene [4], while *B. serrata* samples originating from India are rich in α-thujene [6]. In addition, *B. papyrifera* originating from Ethiopia and Sudan shows notably different chemical profiles compared to the others as octyl acetate is the predominant compound present [7].

**Figure 1:**
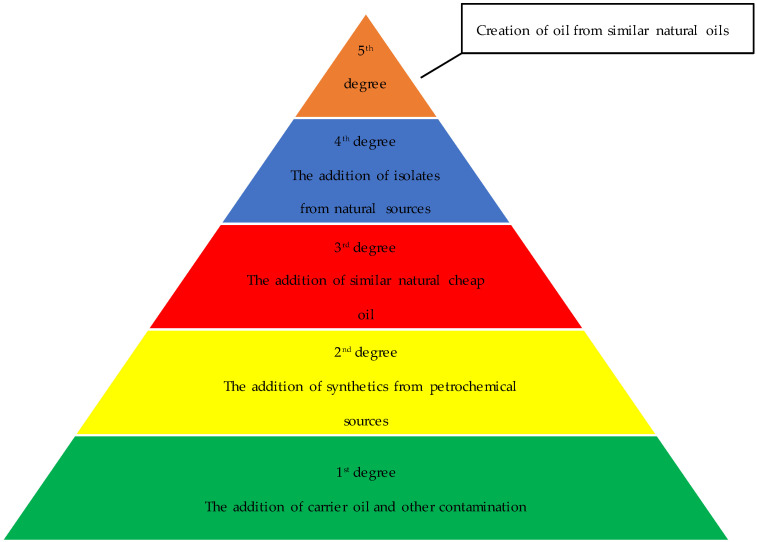
Different degrees of essential oil adulteration.

The essential oil market has grown rapidly in recent years and is a billion-dollar industry due to its reputed health benefits and application in several industries [8]. Therefore, it is a huge challenge for different stakeholders to maintain the quality of essential oils. Different types of adulteration can be observed in the commercial essential oil market. The addition of carrier oils or cooking oils is the most common way of adulterating essential oils [9]. However, more recently, clever ways of adulteration such as the addition of petrochemical-derived synthetic compounds have increased, for example, the addition of synthetic methyl salicylate in wintergreen or birch essential oil, linalool or linalyl acetate to bergamot or lavender oil, and (*E*)-cinnamaldehyde to cinnamon oil [10,11,12]. Similarly, the addition of natural oils with similar compositions is another innovative way of adulteration such as the addition of sweet orange oil to grapefruit oil and wintergreen oil to birch oil. Additionally, adding a natural isolate is a creative way of conducting adulteration (e.g., linalool extracted from ho leaf oil added to lavender or bergamot oil) [11]. Finally, creating an essential oil by combining similar natural oils is a sophisticated and shrewd way of doing an adulteration (e.g., lavender and lemon oil have a similar makeup to bergamot oil and can be used to make bergamot oil (https://www.aromaticplant.org/post/bergamot-adulteration, (accessed on 15 June 2022). Figure 1 presents the different degrees of adulteration in essential oils.

The first and second degrees of adulteration can be detected through marker-based analysis using GC-MS and ^14^C radiocarbon activity [12,13] whereas third to fifth degrees of adulteration are much more complicated to detect as they can easily pass ^14^C radiocarbon activity tests due to the use of natural oil or their isolates. Therefore, we needed to cast an eye over the enantiomeric distributions of different chiral terpenoids present in oil for their purity authentication and species identification [5,14,15]. Thus, chiral gas chromatography-mass spectrometry should always be the choice of a researcher to detect adulterations in essential oils.

In, this research, we analyzed the composition of twenty-one commercial frankincense essential oils and compared them with lab-distilled *Boswellia carteri* essential oil using gas chromatography-mass spectrometry (GC-MS) and chiral GC-MS.

## 2. Results and Discussion

### 2.1. GC-MS Analysis of Commercial and Lab-Distilled Frankincense Essential Oil

The results of the GC-MS analysis of the commercial (F1–F21) and lab-distilled (F22 and F23) frankincense essential oils are presented in the Supplementary File (Table S1), and Table 1 shows the selected constituents. These frankincense essential oils were particularly rich in monoterpene hydrocarbons.

α-Thujene in the commercial samples ranged from 0.4% to 11.6%. Surprisingly, the F3 and F12 samples contained 39.4% and 52.9%, respectively, which was similar to the *Boswellia serrata* species, as previously reported [6]. In the commercial samples, α-pinene ranged from 24.1% to 46.4%, while F3 and F12 showed significantly lower concentrations of α-pinene, 19.1% and 10.6%, respectively, due to the contamination with *B. serrata* as described above. Furthermore, major compounds such as δ-3-carene, limonene, and β-caryophyllene were notably different in samples F3 and F12 compared to the lab distilled samples. Instead, other major compounds such as sabinene, β-pinene, myrcene, and *p*-cymene were in ratios comparable to the lab-distilled F22 and F23.

Ethyl isopropyl phthalate (0.1%) was detected in sample F18, which is generally regarded as a contaminant from plastics [16]. Samples F11, F12, F19, and F20 contained ricinelaidic acid lactone, indicating the addition of castor oil to the frankincense oil [17]. In sample F15, copalic acid was also detected in a trace amount, which indicates the addition of copaiba resin to the frankincense oil [18]. Additionally, the F2, F4, F12, and F17 samples showed the presence of the methyl commate isomer and methyl commate B, which indicates the addition of frankincense resin to frankincense essential oil [19]. Samples F4–F11 along with F19 and F20 contained relatively high percentages of limonene, which might be due to the addition of limonene from petrochemical sources.

Most of the commercial samples, except for F3, F4, F6, F11, F12, and F18, contained either octyl methyl ether or decyl methyl ether, or both, which are generally regarded as contamination from *Boswellia occulta* species, as previously described [1,20,21]. Samples that were not contaminated by *B. occulta* were adulterated in other ways: F3 and F12 were samples of *B. serrata*, F18 contained some other cheap oil, F11 was adulterated using castor oil, F4 was contaminated with frankincense resin, and in F6, synthetic limonene may have been added. Additionally, the F1, F2, F14, F15, and F21 samples contained remarkably high percentages of octyl acetate, which might be due to the addition of synthetic octyl acetate to *B. carteri* essential oils [22]. Finally only two samples, F2 and F7, contained both the biomarker compounds incensole and serratol of *B. carteri*, which were present in the pure lab-distilled samples.

Due to different therapeutic benefits, consumers are using frankincense essential oil in skin care [23] and aromatherapy [3]. Frankincense oil also shows impressive anti-inflammatory and anticancer properties [24,25], but the present study showed that the commercial essential oil market is full of adulterated oils. Therefore, consumers are vulnerable to safety issues of synthetic compounds present in adulterated frankincense oil, as oils are non-compliant with natural labels [26].

### 2.2. Enantiomeric Distribution Analysis of Commercial and Lab-Distilled Frankincense Essential Oils

The results of the CGC-MS analysis of the commercial (F1–F21) and lab-distilled (F22 and F23) frankincense essential oils are presented in Table 2. The enantiomeric distributions of chiral terpenoids were used in a previous study to differentiate the species of *Boswellia* [5]. In this study, we used chiral-GC-MS as a tool for the authentication of commercial frankincense essential oil. To the best of our knowledge, this is the first report on the enantiomeric distribution analysis of chiral terpenoids that was used for the authentication of commercial frankincense essential oils. In both the commercial (F1-F21) and lab-distilled (F22 and F23) frankincense oils, eight chiral terpenoids were detected, except in sample F12, where only seven were detected. The enantiomeric distribution analysis of the lab-distilled samples F22 and F23 showed absolute levorotatory α-thujene and nearly racemic α-pinene and camphene. Additionally, most of the chiral terpenoids were dominated by the levorotatory forms, namely sabinene, β-pinene, limonene, terpinen-4-ol, and α-terpineol.

First, in the F2, F14, and F21 samples, significant changes in the enantiomeric ratios of limonene were observed, while in F1 and F15, both limonene and α-terpineol were changed. Additionally, the GC-MS results of these samples showed a high percentage of octyl acetate. In F3 and F12, the enantiomeric distributions of all chiral terpenoids changed drastically, which is supported by the GC-MS results of these samples with a high percentage of α-thujene from *B. serrata* [6].

Samples F4 and F17 were adulterated with frankincense resin as the GC-MS analysis showed and chiral GC-MS also showed the change in the enantiomeric distributions of β-pinene. From this, we can say that the addition of frankincense resin changes the enantiomeric distributions of β-pinene. Additionally, in the F5–F10 samples, the enantiomeric ratios of limonene changed, in particular, (+)-limonene increased. The GC-MS results of these samples showed an increase in the percentage of limonene compared to those that were lab-distilled. Additionally, in F7, only the enantiomeric ratios of limonene changed while in F10, along with limonene, the enantiomeric ratios of camphene changed and in F5, F6, F8, and F9, the enantiomeric ratios of α-pinene, camphene, and limonene in all three samples changed. From this, it seems that with an increase in the addition of limonene from external sources, the enantiomeric ratios of all chiral terpenes will be altered.

Interestingly, in F11, F19, and F20, the enantiomeric ratios of α-pinene, camphene, and limonene were all changed and the GC-MS results of these samples indicate that they were adulterated with castor oil, along with a high limonene percentage. Furthermore, in F18, the enantiomeric ratios of chiral terpenoids were drastically changed and this was also supported by the GC-MS results, showing an unusual increase in the percentage of some minor compounds that might be due to the addition of some cheap oils. On the other hand, in F13 and F16, although the enantiomeric ratios of all chiral terpenoids were within the ±10 range compared to the lab-distilled samples, the GC-MS results showed that they were contaminated with *B. occulta*. Thus, with the incidental contamination of *B. carteri* by *B. occulta* species, the enantiomeric ratio of chiral terpenoids did not change significantly.

Thus, from the GC-MS and chiral GC-MS analyses, all samples of frankincense essential oil purchased from the market with the claim of pure *Boswellia carteri* were either adulterated or contaminated with other species such as *Boswellia occulta*, and *Boswellia serrata*.

## 3. Materials and Methods

### 3.1. Sample Collection

Oleo-gum-resin of *B. carteri* species from Somaliland was brought by our team to the Aromatic Plant Research Center, Lehi, Utah, USA and distillation was carried out using a Clevenger apparatus as previously described [2]. Additionally, twenty-one commercial frankincense essential oil samples were purchased online in Lehi, Utah, USA from different essential oil brands that claim to be pure *B. carteri*. 

### 3.2. Chemical Composition Analysis by Gas Chromatography-Mass Spectrometry (GC-MS)

Commercial and natural *B. carteri* essential oils were analyzed using a Shimadzu GC-MS-QP2010 Ultra with electron impact (EI) mode with 70 eV along with a ZB-5MS capillary GC column at 40–400 m/z range scans with a scan rate of 3.0 scan/s as previously described [27]. The identification of compounds was carried out through a comparison of the retention indices determined with respect to a homologous series of *n*-alkanes and the comparison of their mass spectra reported in the literature [28] and our own in-house library [29]. The relative percentages of the individual compounds present in frankincense oils are listed in Table 1.

### 3.3. Enantiomeric Distribution Analysis by Chiral Gas Chromatography-Mass Spectrometry (CGC-MS)

A Shimadzu GC-MS-QP2010S with EI mode (70 eV) and B-Dex 325 chiral capillary GC column was used to perform the enantiomeric analysis of commercial and natural frankincense oil as previously described [10]. The percentages of the enantiomers were determined from the peak area. A comparison of the retention times and mass spectral fragmentation patterns with authentic samples obtained from Sigma-Aldrich (Milwaukee, Brookfield, WI, USA) was used to identify the enantiomers. Enantiomeric distributions of different chiral compounds present in the frankincense oils are reported in Table 2.

## 4. Conclusions

Adulteration is a major problem in the essential oil market and has been always a challenge for the researcher in detection. Previously, gas chromatography-mass spectrometry has been widely used for adulteration detection but due to some limitations, it is unable to detect the addition of cheap natural essential oils with similar composition. To the best of our knowledge, for the first time, we compared the enantiomeric distributions of chiral terpenoids present in commercial and lab-distilled frankincense essential oils for authentication. Interestingly, there is a drastic change in the enantiomeric distributions of chiral terpenoids if there is any kind of natural or synthetic adulteration in the frankincense essential oil. Thus, with the application of gas chromatography-mass spectrometry and chiral gas chromatography-mass spectrometry, different degrees of adulteration can be readily detected in frankincense essential oils.

## Figures and Tables

**Table 1:** The selected constituents of the commercial and lab-distilled frankincense (*B. carteri*) essential oils.

R.I	Compounds	F1	F2	F3	F4	F5	F6	F7	F8	F9	F10	F11	F12	F13	F14	F15	F16	F17	F18	F19	F20	F21	F22	F23
924	α-Thujene	5.6	11.0	39.4	2.2	0.4	9.5	3.4	7.3	9.2	6.7	8.9	52.9	3.4	11.1	5.5	9.0	4.4	11.6	9.4	11.0	5.4	9.1	9.2
931	α-Pinene	40.3	37.2	19.1	35.5	37.9	29.0	46.4	29.2	29.8	27.8	29.8	10.6	31.0	38.8	41.9	24.1	31.1	30.6	27.4	41.2	36.8	31.8	33.3
948	Camphene	0.4	0.6	0.2	1.1	1.7	1.5	1.2	1.3	1.4	1.0	1.4	0.2	0.8	0.4	0.4	0.4	0.9	0.7	1.5	1.4	0.4	0.7	0.8
972	Sabinene	6.8	4.6	4.0	4.4	5.6	4.9	3.2	2.7	5.7	4.2	4.6	8.0	3.7	4.5	6.4	5.1	3.9	4.2	5.6	5.8	2.9	3.5	3.2
978	β-Pinene	5.3	3.9	5.1	2.0	3.7	2.9	4.5	1.8	3.5	2.3	2.7	0.7	1.6	3.8	4.9	3.0	1.7	2.3	2.9	3.7	3.9	2.1	2.4
989	Myrcene	1.5	3.5	0.7	5.3	6.5	4.8	3.4	3.6	4.6	3.2	4.5	1.4	4.2	2.3	1.5	3.7	7.1	0.5	5.0	3.3	1.9	3.2	3.4
1008	α-Phellandrene	2.4	2.6	1.9	4.1	4.3	3.0	1.8	2.2	2.3	1.9	2.8	2.5	2.1	1.8	2.3	2.2	2.6	t	2.3	0.7	0.8	1.6	1.4
1009	δ-3-Carene	1.6	0.9	5.8	2.6	0.8	1.4	0.8	1.4	1.0	2.4	1.4	5.8	0.9	0.7	1.5	1.4	0.6	3.1	0.9	1.0	0.6	0.9	0.7
1024	*p*-Cymene	3.6	2.4	6.1	5.5	5.3	5.2	4.5	4.9	4.9	4.4	5.0	3.3	3.7	3.1	3.3	4.3	4.5	10.1	5.5	4.9	3.0	5.5	5.6
1027	Octyl methyl ether	-	0.1	-	-	-	-	0.2	-	0.1	-	-	-	0.7	t	-	-	t	-	-	0.3	-	-	-
1028	Limonene	8.6	10.4	3.9	18.6	21.0	25.5	18.2	23.2	26.0	21.4	26.1	3.0	12.3	10.4	8.1	14.3	14.2	5.4	25.6	17.3	12.4	10.1	10.1
1195	α-Terpineol	0.5	0.3	0.2	0.5	0.1	0.2	0.4	0.6	0.2	0.6	0.2	t	0.5	0.3	0.5	0.4	0.4	2	0.1	0.2	0.3	0.8	0.8
1198	Methyl chavicol	t	0.1	1.8	-	-	0.2	0.1	0.1	0.3	0.2	0.2	2.2	-	0.1	t	0.1	-	3.8	0.3	0.1	0.1	-	-
1209	Octyl acetate	4.2	5.2	-	-	t	-	0.3	0.5	0.3	0.6	t	-	0.1	4.7	3.9	0.1	0.1	-	0.1	0.4	7.7	0.9	1.0
1228	Decyl methyl ether	0.2	0.3	-	-	t	-	0.7	t	0.4	0.2	-	-	3.0	t	0.2	t	0.1	-	0.1	1.2	0.1	-	-
1381	β-Bourbonene	0.1	0.1	0.3	0.1	t	0.1	0.2	0.1	0.2	0.2	0.1	0.6	0.5	0.1	0.1	0.2	0.1	2.1	0.1	0.2	0.1	0.3	0.3
1417	(*E*)-β-Caryophyllene	7.2	4.1	t	2.7	4.6	1.3	0.8	2.9	1.2	2.6	1.3	-	3.9	4.2	6.8	4	3.3	0.4	1.9	0.7	5.8	1.8	1.9
1585	Ethyl isopropyl phthalate	-	-	-	-	-	-	-	-	-	-	-	-	-	-	-	-	-	0.1	-	-	-	-	-
2057	Ricinelaidic acid lactone	-	-	-	-	-	-	-	-	-	-	t	t	-	-	-	-	-	-	t	0.1	-	-	-
2145	Serratol	1.0	0.5	t	-	-	0.1	0.3	-	-	-	0.2	0.1	2.1	1.6	1.6	1.7	0.3	1.3	0.2	0.1	1.7	0.7	0.7
2146	Incensole	-	0.1	-	-	-	-	0.1	-	-	-	-	-	-	-	-	-	-	-	-	-	-	0.2	0.1
2149	Incensyl acetate	t	0.2	-	t	t	t	-	t	-	-	-	-	-	0.3	-	t	-	-	t	-	-	-	-
2330	Copalic acid	-	-	-	-	-	-	-	-	-	-	-	-	-	-	t	-	-	-	-	-	-	-	-
2678	Methyl commate isomer	-	-	-	-	-	-	-	-	-	-	-	-	-	-	-	-	7.1	-	-	-	-	-	-
2792	Methyl commate B	-	0.9	-	0.1	-	-	-	-	-	-	-	0.3	-	-	-	-	-	-	-	-	-	-	-

“-” indicates not detected and “t” indicates trace (≤0.05%).

**Table 2:** The enantiomeric distribution of chiral terpenoids present in the commercial and lab-distilled *B. carteri* essential oils.

Chiral Compounds	F1	F2	F3	F4	F5	F6	F7	F8	F9	F10	F11	F12	F13	F14	F15	F16	F17	F18	F19	F20	F21	F22	F23
α-Thujene	(+) 0.0: (–) 100.0	(+) 0.0: (–) 100.0	(+) 100.0: (–) 0.0	(+) 0.0: (–) 100.0	(+) 11.4: (–) 88.6	(+) 0.0: (–) 100.0	(+) 0.0: (–) 100.0	(+) 0.0: (–) 100.0	(+) 0.0: (–) 100.0	(+) 0.0: (–) 100.0	(+) 0.0: (–) 100.0	(+) 100.0: (–) 0.0	(+) 0.0: (–) 100.0	(+) 0.0: (–) 100.0	(+) 7.6: (–) 92.4	(+) 0.0: (–) 100.0	(+) 0.0: (–) 100.0	(+) 0.0: (–) 100.0	(+) 0.0: (–) 100.0	(+) 0.0: (–) 100.0	(+) 0.0: (–) 100.0	(+) 0.0: (–) 100.0	(+) 0.0: (–) 100.0
α-Pinene	(+) 53.3: (–) 46.7	(+) 54.3: (–) 45.7	(+) 77.9: (–) 22.1	(+) 41.2: (–) 58.8	(+) 26.5: (–) 73.5	(+) 28.4: (–) 71.6	(+) 40.3: (–) 59.7	(+) 31.9: (–) 68.1	(+) 31.6: (–) 68.4	(+) 36.5: (–) 63.5	(+) 31.0: (–) 69.0	(+) 64.8: (–) 35.2	(+) 45.7: (–) 54.3	(+) 52.7: (–) 47.3	(+) 53.2: (–) 46.8	(+) 42.1: (–) 57.9	(+) 47.0: (–) 53.0	(+) 88.5: (–) 11.5	(+) 28.6: (–) 71.4	(+) 25.3: (–) 74.7	(+) 50.8: (–) 49.2	(+) 44.4: (–) 55.6	(+) 42.1: (–) 57.9
Camphene	(+) 40.3: (–) 59.7	(+) 43.9: (–) 56.1	(+) 33.0: (–) 67.0	(+) 46.3: (–) 53.7	(+) 29.1: (–) 70.9	(+) 31.3: (–) 68.7	(+) 34.4: (–) 65.6	(+) 30.4: (–) 69.6	(+) 30.1: (–) 69.9	(+) 28.9: (–) 71.1	(+) 30.8: (–) 69.2	(+) 100.0: (–) 0.0	(+) 52.7: (–) 47.3	(+) 46.1: (–) 53.9	(+) 39.5: (–) 60.5	(+) 46.8: (–) 53.2	(+) 53.2: (–) 46.8	(+) 60.5: (–) 39.5	(+) 27.7: (–) 72.3	(+) 24.4: (–) 75.6	(+) 47.4: (–) 52.6	(+) 42.2: (–) 57.8	(+) 45.6: (–) 54.4
Sabinene	(+) 7.2: (–) 92.8	(+) 13.3: (–) 86.7	(+) 19.6: (–) 80.4	(+) 7.5: (–) 92.5	(+) 6.1: (–) 93.9	(+) 8.1: (–) 91.9	(+) 15.2: (–) 84.8	(+) 10.3: (–) 89.7	(+) 10.2: (–) 89.8	(+) 20.0: (–) 80.0	(+) 7.0: (–) 93.0	(+) 20.0: (–) 80.0	(+) 20.9: (–) 79.1	(+) 9.4: (–) 90.6	(+) 7.4: (–) 92.6	(+) 14.7: (–) 85.3	(+) 8.9: (–) 91.1	(+) 46.9: (–) 53.1	(+) 8.4: (–) 91.6	(+) 11.6: (–) 88.4	(+) 9.5: (–) 90.5	(+) 16.3: (–) 83.7	(+) 16.8: (–) 83.2
β-Pinene	(+) 7.3: (–) 92.7	(+) 10.5: (–) 89.5	(+) 4.7: (–) 95.3	(+) 33.0: (–) 67.0	(+) 6.6: (–) 93.4	(+) 8.8: (–) 91.2	(+) 8.4: (–) 91.6	(+) 15.4: (–) 84.6	(+) 8.5: (–) 91.5	(+) 14.9: (–) 85.1	(+) 14.6: (–) 85.4	(+) 19.2: (–) 80.8	(+) 27.4: (–) 72.6	(+) 7.9: (–) 92.1	(+) 7.5: (–) 92.5	(+) 9.7: (–) 90.3	(+) 30.2: (–) 69.8	(+) 14.0: (–) 86.0	(+) 8.2: (–) 91.8	(+) 8.4: (–) 91.6	(+) 7.4: (–) 92.6	(+) 17.4: (–) 82.6	(+) 15.0: (–) 85.0
Limonene	(+) 40.4: (–) 59.6	(+) 45.9: (–) 54.1	(+) 71.1: (–) 28.9	(+) 11.3: (–) 88.7	(+) 76.3: (–) 23.7	(+) 35.3: (–) 64.7	(+) 53.2: (–) 46.8	(+) 41.6: (–) 58.4	(+) 35.3: (–) 64.7	(+) 44.3: (–) 55.7	(+) 41.6: (–) 58.4	(+) 84.7: (–) 15.3	(+) 23.0: (–) 77.0	(+) 36.9: (–) 63.1	(+) 40.6: (–) 59.4	(+) 22.0: (–) 78.0	(+) 15.8: (–) 84.2	(+) 90.5: (–) 9.5	(+) 33.3: (–) 66.7	(+) 56.4: (–) 43.6	(+) 44.7: (–) 55.3	(+) 16.5: (–) 83.5	(+) 15.2: (–) 84.8
Terpinen-4-ol	(+) 31.0: (–) 69.0	(+) 33.3: (–) 66.7	(+) 25.9: (–) 74.1	(+) 28.9: (–) 71.1	(+) 29.7: (–) 70.3	(+) 28.4: (–) 71.6	(+) 38.1: (–) 61.9	(+) 29.2: (–) 70.8	(+) 34.6: (–) 65.4	(+) 36.3: (–) 63.7	(+) 30.0: (–) 70.0	(+) 29.4: (–) 70.6	(+) 34.1: (–) 65.9	(+) 31.1: (–) 68.9	(+) 30.7: (–) 69.3	(+) 27.0: (–) 73.0	(+) 29.0: (–) 71.0	(+) 28.2: (–) 71.8	(+) 28.9: (–) 71.1	(+) 39.7: (–) 60.3	(+) 32.4: (–) 67.6	(+) 31.1: (–) 68.9	(+) 29.8: (–) 70.2
α-Terpineol	(+) 17.4: (–) 82.6	(+) 33.9: (–) 66.1	(+) 37.1: (–) 62.9	(+) 31.4: (–) 68.6	(+) 31.3: (–) 68.7	(+) 38.1: (–) 61.9	(+) 40.1: (–) 59.9	(+) 34.0: (–) 66.0	(+) 38.5: (–) 61.5	(+) 34.8: (–) 65.2	(+) 30.4: (–) 69.6	nd	(+) 34.0: (–) 66.0	(+) 30.6: (–) 69.4	(+) 16.5: (–) 83.5	(+) 28.6: (–) 71.4	(+) 34.1: (–) 65.9	(+) 57.3: (–) 42.7	(+) 32.7: (–) 67.3	(+) 33.4: (–) 66.6	(+) 30.3: (–) 69.7	(+) 32.2: (–) 67.8	(+) 31.1: (–) 68.9

“–” indicates levorotatory; “+” indicates dextrorotatory; “nd” indicates not detected.

## Data Availability

Not applicable.

## Supplementary Materials

The following supporting information can be downloaded at: https://www.mdpi.com/article/10.3390/plants11162134/s1, Table S1: The compositional analysis of commercial and lab-distilled frankincense (*B. carteri*) essential oils.
