# Supplementary material for: Comparison of Volatile Constituents Present in Commercial and Lab-Distilled Frankincense (Boswellia carteri) Essential Oils for Authentication
Source: Plants (Basel). 2022 Aug 16;11(16):2134. doi: 10.3390/plants11162134 (PMC9415502; doi:10.3390/plants11162134)
Supplement: Supplementary file 1 [file plants-11-02134-s001.zip › plants-1852616-supplementary.pdf]

Table S1: Compositional analysis of commercial and lab distilled frankincense (*B. carteri*) essential oils.

R.I	Compounds	F1	F2	F3	F4	F5	F6	F7	F8	F9	F10	F11	F12	F13	F14	F15	F16	F17	F18	F19	F20	F21	F22	F23
916	Hashishene	0.1	0.2	0.1	0.1	t	t	0.2	0.2	0.2	0.4	t	0.1	0.2	0.1	0.1	0.1	0.2	t	t	0.2	t	0.4	0.4
921	Tricyclene	0.1	0.1	0.1	0.1	0.1	0.1	0.1	0.1	0.1	0.1	0.1	t	t	0.1	0.1	t	0.1	0.2	0.1	0.1	0.1	0.1	0.1
924	α-Thujene	5.6	11.0	39.4	2.2	0.4	9.5	3.4	7.3	9.2	6.7	8.9	52.9	3.4	11.1	5.5	9.0	4.4	11.6	9.4	11.0	5.4	9.1	9.2
931	α-Pinene	40.3	37.2	19.1	35.5	37.9	29.0	46.4	29.2	29.8	27.8	29.8	10.6	31.0	38.8	41.9	24.1	31.1	30.6	27.4	41.2	36.8	31.8	33.3
942	Thujadiene	0.1	0.1	0.4	0.1	t	t	t	0.1	0.1	t	0.1	0.3	0.1	0.1	0.1	0.3	0.1	t	0.1	t	0.1	0.6	0.7
945	α-Fenchene	t	t	t	t	0.2	0.2	0.1	0.1	0.2	0.1	0.2	t	t	t	t	t	t	t	0.2	0.2	t	t	t
948	Camphene	0.4	0.6	0.2	1.1	1.7	1.5	1.2	1.3	1.4	1.0	1.4	0.2	0.8	0.4	0.4	0.4	0.9	0.7	1.5	1.4	0.4	0.7	0.8
954	Thuja-2,4(10)-diene	0.1	0.1	t	0.5	0.1	0.2	0.3	0.6	0.2	0.6	0.3	t	0.4	0.1	0.1	0.1	0.3	t	0.1	0.2	0.1	0.7	0.8
970	3,7,7-Trimethyl-1,3,5-cycloheptatriene	t	t	t	0.1	t	0.1	t	0.2	-	0.3	0.1	t	0.2	t	t	t	0.1	-	-	t	t	0.1	0.1
972	Sabinene	6.8	4.6	4.0	4.4	5.6	4.9	3.2	2.7	5.7	4.2	4.6	8.0	3.7	4.5	6.4	5.1	3.9	4.2	5.6	5.8	2.9	3.5	3.2
978	β-Pinene	5.3	3.9	5.1	2.0	3.7	2.9	4.5	1.8	3.5	2.3	2.7	0.7	1.6	3.8	4.9	3.0	1.7	2.3	2.9	3.7	3.9	2.1	2.4
980	*trans*-Isolimonene	t	t	-	-	0.1	0.1	0.1	t	0.1	t	0.1	t	-	t	t	-	-	-	0.1	0.1	t	-	-
986	3-*p*-Menthene	-	-	t	-	t	t	-	t	t	-	t	-	-	-	-	-	-	-	t	-	-	-	-
989	Myrcene	1.5	3.5	0.7	5.3	6.5	4.8	3.4	3.6	4.6	3.2	4.5	1.4	4.2	2.3	1.5	3.7	7.1	0.5	5.0	3.3	1.9	3.2	3.4
992	*trans*-Menthane	-	-	-	-	-	-	-	-	-	-	t	-	-	-	-	-	-	-	t	t	-	-	-
998	2-*p*-Menthene	-	-	t	-	t	t	t	-	t	-	t	-	-	-	-	-	-	-	t	-	-	-	-
1003	*para*-Mentha-1(7),8-diene	t	t	t	t	0.4	0.3	0.3	0.1	0.4	0.1	0.3	t	t	0.1	t	t	t	t	0.3	0.3	0.1	-	-
1007	3-Ethenyl-1,2-dimethyl-1,4-cyclohexadiene	-	-	-	0.1	-	-	-	0.2	-	0.1	-	-	-	-	-	-	-	-	-	-	-	0.2	0.2
1008	α-Phellandrene	2.4	2.6	1.9	4.1	4.3	3.0	1.8	2.2	2.3	1.9	2.8	2.5	2.1	1.8	2.3	2.2	2.6	t	2.3	0.7	0.8	1.6	1.4
1009	δ-3-Carene	1.6	0.9	5.8	2.6	0.8	1.4	0.8	1.4	1.0	2.4	1.4	5.8	0.9	0.7	1.5	1.4	0.6	3.1	0.9	1.0	0.6	0.9	0.7
1015	1,4-Cineole	t	-	t	-	t	t	-	t	t	t	t	t	-	t	t	-	-	t	t	t	-	-	-
1016	α-Terpinene	0.2	0.1	1.1	0.4	0.6	1.4	0.6	1.2	1	1.1	1.4	0.3	0.2	0.3	0.2	0.4	0.2	-	0.9	0.1	0.2	0.7	0.7
1019	*m*-Cymene	t	t	0.1	t	t	t	t	t	t	t	t	0.1	t	t	t	t	t	0.2	t	t	t	0.1	0.1
1024	*p*-Cymene	3.6	2.4	6.1	5.5	5.3	5.2	4.5	4.9	4.9	4.4	5.0	3.3	3.7	3.1	3.3	4.3	4.5	10.1	5.5	4.9	3.0	5.5	5.6
1026	2-Acetyl-3-methylfuran	t	t	0.1	-	-	t	-	-	-	-	-	0.4	-	t	-	0.1	t	0.2	-	-	-	-	t
1027	Octyl methyl ether	-	0.1	-	-	-	-	0.2	-	0.1	-	-	-	0.7	t	-	-	t	-	-	0.3	-	-	-
1028	Limonene	8.6	10.4	3.9	18.6	21.0	25.5	18.2	23.2	26.0	21.4	26.1	3.0	12.3	10.4	8.1	14.3	14.2	5.4	25.6	17.3	12.4	10.1	10.1
1029	β-Phellandrene	0.1	0.3	0.7	0.4	0.1	0.1	0.2	0.3	0.2	t	-	0.7	0.5	0.2	t	-	0.1	0.1	0.3	0.2	0.1	0.4	0.4
1031	1,8-Cineole	0.3	0.3	0.1	0.2	t	0.1	0.2	t	t	0.1	-	0.1	0.2	0.4	0.2	0.9	t	0.2	0.1	0.2	0.3	0.3	0.3

1034	(*Z*)-β-Ocimene	t	0.3	0.2	0.1	t	0.1	0.1	0.2	0.1	0.4	0.1	0.7	0.1	0.1	t	0.1	0.2	0.1	0.1	0.1	0.1	0.3	0.3
1046	(*E*)-β-Ocimene	t	0.1	0.2	0.1	t	t	0.1	0.1	0.1	0.1	0.1	0.3	0.1	0.1	t	0.1	0.1	t	0.1	t	t	0.1	0.1
1058	γ-Terpinene	0.3	0.2	2.7	0.6	0.3	0.9	0.5	1.0	0.6	1.0	0.9	0.6	0.3	0.4	0.3	0.8	0.3	0.2	0.5	0.2	0.3	1.2	1.1
1069	1-Octanol	-	-	-	-	-	-	t	-	-	-	-	-	-	0.3	0.1	-	-	-	-	t	-	0.1	0.2
1071	*cis*-Sabinene hydrate	t	t	t	t	-	t	-	-	t	t	t	t	t	-	t	t	t	0.3	t	-	t	-	-
1073	Pinol	t	t	t	t	-	t	t	0.1	t	0.1	t	t	t	-	-	t	t	-	t	t	0.1	t	0.1
1075	2-Decyl methyl ether	-	-	-	-	-	-	-	-	-	-	-	-	t	-	-	-	-	-	-	t	-	-	-
1083	*p*-Mentha-2,4(8)-diene	-	-	t	-	-	-	-	-	-	-	-	t	-	-	-	-	-	-	-	-	-	0.3	-
1088	Terpinolene	0.1	0.1	2.5	0.2	0.1	0.5	0.2	0.5	0.4	0.4	0.6	0.4	0.1	0.1	0.1	0.1	0.1	0.1	0.4	0.1	0.1		0.3
1092	*p*-Cymenene	t	t	t	0.1	t	0.2	0.1	0.5	0.1	0.4	0.2	t	0.1	0.1	0.1	0.1	0.1	t	t	0.1	0.1	0.3	0.3
1095	*cis-p*-Menthenyl methyl ether	-	-	t	-	-	-	-	-	-	-	-	-	-	-	-	-	-	0.1	-	-	-	-	-
1096	α-Pinene oxide	0.1	-	-	-	-	-	-	t	-	t	t	t	-	0.1	0.1	-	-	-	-	0.2	-	-	-
1098	Perillene	t	t	-	0.1	-	t	t	0.1	-	t	-	-	0.1	-	-	t	0.1	-	t	t	t	0.1	0.1
1099	Linalool	-	0.3	0.1	0.2	-	-	0.1	0.1	-	0.1	t	0.3	0.1	0.2	t	0.1	0.2	0.6	0.1	-	-	0.1	0.1
1102	*trans*-Sabinene hydrate	-	-	t	-	-	-	-	-	-	-	-	-	-	-	-	-	-	0.3	-	-	-	t	-
1108	*cis*-Thujone	-	-	t	t	-	-	-	-	-	t	-	t	-	-	-	-	0.1	0.3	-	-	-	t	t
1117	*trans*-Thujone	t	t	0.1	-	-	t	-	t	t	-	t	0.3	0.1	0.1	t	0.2	-	1.1	t	t	t	0.4	0.4
1118	3-Octyl acetate	-	-	-	t	-	-	-	t	-	t	-	-		-	-	-	-	-	-	-	-	-	-
1119	Myrcenol	-	t	-	t	-	-	-	t	-	t	-	-	0.1	-	-	t	0.1	-	-	-	-	0.1	t
1122	*trans-p*-Mentha-2,8-dien-1-ol	t	t	-	t	-	-	-	-	-	t	-	-	t	t	t	-	t	t	t	t	t	t	t
1124	*cis-p*-Menth-2-en-1-ol	-	-	t	t	-	-	-	-	-	t	t	t	t	-	-	t	t	-	-	-	-	0.1	t
1126	α-Campholenal	t	0.1	-	0.3	t	0.1	0.1	0.2	0.1	0.3	0.1	-	0.4	t	t	0.1	0.2	0.1	t	-	t	0.3	0.3
1128	Methyl nonyl ether	-	-	-	-	-	-	-	-	-	-	-	-	-	-	-	-	-	-	-	0.1	-	-	-
1129	4,5-Epoxy-*trans*-carene	-	-	-	t	-	t	-	t	-	0.1	t	-	t	-	-	t	-	-	-	-	-	0.1	0.1
1131	Terpin-3-en-1-ol	-	-	t	-	-	-	-	-	-	-	-	-	-	-	-	-	-	0.1	-	-	-	-	-
1133	*cis*-Limonene oxide	-	-	t	t	t	t	-	-	t	t	t	t	-	t	-	-	t	t	t	t	-	-	-
1137	*cis-p*-Mentha-2,8-dien-1-ol	-	t	-	-	-	-	-	-	-	-	-	-	t	t	t	t	t	-	t	t	t	t	-
1138	*trans*-Sabinol	-	-	-	-	-	-	-	-	-	-	-	t	-	-	-	-	-	-	-	-	-	0.1	0.1
1140	*trans*-Pinocarveol	t	0.1	-	0.2	t	t	0.2	0.1	t	0.2	0.1	-	0.7	0.1	0.1	0.2	0.1	-	t	0.2	t	0.8	0.8
1142	*trans-p*-Menth-2-en-1-ol	-	-	-	0.1	-	-	-	-	-	0.1	-	-	-	t	-	0.1	0.3	-	-	-	-	-	-
1143	Epoxyterpinolene	-	-	0.1	-	-	t	-	-	t	-	t	t	-	-	-	-	-	0.2	t	t	-	-	-
1145	Camphor	-	-	t	-	-	t	-	t	-	0.1	t	t	-	-	-	-	0.2	-	-	-	-	t	t
1146	*trans*-Verbenol	-	t	-	-	-	-	0.1	-	-	-	-	-	0.6	t	0.1	0.2	-	-	-	0.1	-	0.4	0.5
1149	α-Phellandren-8-ol	t	0.1	-	0.2	t	t	0.1	0.1	0.1	0.3	t	-	0.3	0.1	0.1	0.1	0.4	-	t	0.1	0.1	0.3	0.3

1153	Sabina ketone	-	-	-	t	-	-	-	-	-	t	-	-	t	-	-	t	t	t	-	t	-	t	t
1158	β-Pinene oxide	-	-	-	-	-	-	-	-	-	-	-	-	-	-	-	-	-	-	-	-	-	0.1	0.1
1159	*trans*-Pinocamphone	t	t	-	0.1	t	t	0.1	0.1	0.1	0.2	t	-	0.1	t	t	t	0.1	-	t	t	t	0.1	0.1
1161	*cis*-Chrysanthenol	-	t	t	0.1	-	-	-	-	-	0.1	-	-	t	-	-	t	-	-	-	-	-	-	-
1163	Pinocarvone	-	-	-	-	-	t	t	0.1	t	-	t	-	t	-	-	-	-	-	-	t	-	0.1	0.1
1168	α-Phellandrene epoxide	-	-	-	-	-	-	-	-	-	-	-	0.1	-	-	-	-	-	-	-	t	-	0.2	0.2
1169	Umbellulone	-	-	-	-	-	-	-	-	-	-	-	-	-	-	-	-	t	0.1	-	-	-	-	-
1169	*endo-iso*-Camphone	t	t	-	t	t	t	-	t	t	t	t	t	t	t	t	t	-	0.1	t	t	t	-	-
1170	Borneol	-	-	t	-	-	-	-	-	t	-	-	-	t	t	-	-	t	-	t	-	-	t	t
1171	*p*-Mentha-1,5-dien-8-ol	0.1	0.3	t	0.4	t	0.1	0.2	0.3	0.1	0.7	0.1	-	0.6	0.1	0.1	0.3	0.3	-	t	-	0.1	0.9	1.0
1173	*exo-iso*-Camphone	t	t	-	0.1	-	t	-	0.1	-	-	t	-	-	-	-	-	-	-	-	-	0.1	-	-
1176	Verbenyl ethyl ether	-	-	-	-	t	-	-	-	-	-	-	-	-	-	-	-	-	0.2	-	-	-	-	-
1179	*p*-1,8-Menthadien-4-ol	-	-	-	t	-	-	-	-	-	-	-	-	t	-	t	t	t	-	-	-	-	t	t
1180	Terpinen-4-ol	1.0	0.4	1.0	0.7	0.2	0.5	0.5	0.9	0.5	1.1	0.5	0.7	0.7	0.5	1.0	0.9	0.5	2.4	0.5	0.3	0.5	1.5	1.5
1183	Thuj-3-en-10-al	-	-	-	-	-	-	-	-	-	-	-	t	-	-	-	t	-	-	-	-	-	t	t
1184	*p*-Methylacetophenone	-	-	-	t	-	-	-	t	-	-	-	-	-	-	-	-	t	-	-	t	-	t	t
1187	*p*-Cymen-8-ol	t	0.1	t	0.1	t	t	t	t	0.1	0.1	t	0.1	0.2	0.1	0.1	0.1	0.1	0.5	t	0.1	0.1	0.2	0.2
1194	Myrtenol	-	-	-	-	-	-	-	-	-	-	-	-	-	-	-	-	-	-	-	-	-	t	t
1195	α-Terpineol	0.5	0.3	0.2	0.5	0.1	0.2	0.4	0.6	0.2	0.6	0.2	t	0.5	0.3	0.5	0.4	0.4	2	0.1	0.2	0.3	0.8	0.8
1198	Methyl chavicol	t	0.1	1.8	-	-	0.2	0.1	0.1	0.3	0.2	0.2	2.2	-	0.1	t	0.1	-	3.8	0.3	0.1	0.1	-	-
1200	γ-Terpineol	-	-	-	-	-	-	-	-	-	-	-	-	-	-	-	-	-	0.2	-	-	-	-	-
1202	*cis*-Sabinol	-	-	-	-	-	-	t	-	-	-	t	0.1	-	t	t	0.1	-	-	-	t	-	0.1	0.1
1206	Verbenone	0.1	0.2	-	0.3	t	0.1	0.1	0.2	0.1	0.4	0.1	t	0.4	0.1	0.1	0.2	0.3	0.1	0.1	0.2	0.2	0.5	0.5
1209	Octyl acetate	4.2	5.2	-	-	t	-	0.3	0.5	0.3	0.6	t	-	0.1	4.7	3.9	0.1	0.1	-	0.1	0.4	7.7	0.9	1.0
1218	*trans*-Carveol	-	-	-	-	-	-	t	-	-	-	-	-	0.1	t	t	t	-	-	-	t	-	0.2	0.2
1228	Decyl methyl ether	0.2	0.3	-	-	t	-	0.7	t	0.4	0.2	-	-	3.0	t	0.2	t	0.1	-	0.1	1.2	0.1	-	-
1242	Carvone	t	0.1	t	0.1	t	0.1	t	0.2	t	0.1	0.1	-	0.2	0.1	t	0.1	0.1	t	t	t	0.1	0.2	0.2
1243	Cuminaldehyde	t	t	t	t	t	t	t	0.1	t	0.1	t	-	-	t	-	t	t	t	t	t	t	0.1	0.1
1248	Carvotanacetone	t	t	-	t	t	0.1	t	0.1	t	t	0.1	-	t	0.1	t	-	t	-	-	t	0.1	0.1	-
1252	Linalyl acetate	-	-	t	-	-	-	-	-	-	-	-	0.1	-	-	-	0.1	-	0.3	t	-	-	-	-
1253	Piperitone	-	t	-	t	-	-	-	-	-	-	-	-	-	-	-	-	-	-	-	-	-	-	0.1
1265	3,5-Dimethoxytoluene	t	t	-	0.1	t	t	t	t	t	0.1	-	-	0.1	-	-	t	0.1	-	t	t	t	0.1	0.1
1276	*trans*-Ascaridol glycol	-	-	-	-	-	t	-	-	-	-	-	t	-	-	-	-	-	0.1	t	t	-	-	-
1280	Phellandral	t	t	-	t	-	t	t	0.1	t	0.1	t	-	t	t	t	t	t	-	t	-	t	0.1	t

1282	Bornyl acetate	0.1	0.3	t	0.5	t	0.1	0.2	0.4	0.1	0.4	0.1	t	0.4	0.2	0.1	0.3	0.3	0.1	0.1	0.1	0.2	0.5	0.5
1288	Isobornyl acetate	-	-	0.1	-	-	-	-	-	-	-	-	-	-	-	-	-	-	-	-	-	-	-	-
1295	1,2:8,9-Diepoxy-*p*-menthane	-	-	-	-	-	-	-	-	-	-	-	-	-	-	-	-	-	-	-	0.1	-	-	-
1296	Carvacrol	t	t	t	t	t	t	t	0.1	t	0.1	t	t	0.1	t	t	0.1	t	0.2	-	-	t	0.1	0.1
1326	2-(1-Methyl-2-oxopropyl)-cyclohexanone	t	-	t	t	t	-	-	-	t	-	-	0.1	-	-	t	-	-	-	t	0.1	t	-	-
1331	Bicycloelemene	t	t	-	t	t	t	-	t	t	t	t	-	t	-	t	t	t	0.1	t	-	t	-	t
1346	α-Terpinyl acetate	-	-	t	-	-	-	-	-	-	-	-	-	-	-	-	-	-	0.3	-	-	-	-	-
1348	α-Cubebene	0.1	0.1	-	0.2	-	0.1	t	0.1	0.1	0.1	0.1	0.1	0.4	0.1	0.1	0.3	0.2	-	0.1	t	0.2	0.3	0.3
1367	Cyclosativene	-	-	-	-	-	t	t	-	t	-	-	t	0.1	t	t	t	-	-	t	-	-	-	-
1368	α-Ylangene	t	t	t	t	t	-	-	t	-	t	-	t	t	t	t	t	0.1	0.1	-	-	t	0.1	0.1
1374	α-Copaene	0.6	0.4	0.1	0.6	0.4	0.2	0.2	0.5	0.2	0.4	0.2	0.1	1.0	0.5	0.6	0.9	0.9	0.7	0.4	0.2	0.9	0.5	0.6
1376	Geranyl acetate	-	t	-	-	-	-	-	t	t	0.1	-	-	-	-	-	-	-	-	-	-	-	-	-
1381	β-Bourbonene	0.1	0.1	0.3	0.1	t	0.1	0.2	0.1	0.2	0.2	0.1	0.6	0.5	0.1	0.1	0.2	0.1	2.1	0.1	0.2	0.1	0.3	0.3
1383	α-Bourbonene	-	-	t	-	-	-	t	-	t	t	-	t	t	-	-	-	-	0.2	-	t	-	-	-
1386	β-Cubebene	t	t	-	t	t	-	-	-	-	-	t	-	0.1	t	t	0.1	0.1	-	t	-	0.1	0.1	0.1
1388	β-Elemene	0.4	0.4	-	1.1	0.2	0.2	0.2	0.7	0.2	0.4	0.2	-	1.4	0.6	0.4	0.5	0.6	0.1	0.3	t	0.6	0.4	0.3
1399	β-Longipinene	-	-	-	-	-	-	-	-	-	-	-	0.1	-	-	-	-	-	0.3	-	-	-	-	-
1404	Methyl eugenol	-	-	0.1	-	-	t	-	-	t	-	-	0.1	-	-	-	-	-	0.5	t	-		-	-
1405	(*Z*)-β-Caryophyllene	t	t	-	t	-	-	-	t	t	t	-	-	t	t	t	t	t	-	-	-	t	-	-
1406	β-Maaliene	t	t	-	t	t	t	-	t	-	-	-	t	0.1	t	t	0.1		-	t	-	t	-	-
1406	α-Gurjunene	-	-	-	-	-	-	-	-	t	t	t	-	-	-	-	-	0.1	-	-	-	-	0.1	0.1
1417	(*E*)-β-Caryophyllene	7.2	4.1	t	2.7	4.6	1.3	0.8	2.9	1.2	2.6	1.3	-	3.9	4.2	6.8	4	3.3	0.4	1.9	0.7	5.8	1.8	1.9
1419	β-Ylangene	-	-	t	-	-	-	-	-	-	-	-	0.1	-	-	-	-	-	0.2	-	-	-	-	-
1430	γ-Elemene	-	-	-	-	0.1	t	0.1	-	0.1	-	t	-	-	-	t	-	-	-	0.1	t	t	-	-
1431	β-Copaene	t	t	t	t	-	-	-	t	-	t	-	0.1	0.1	t	-	0.1	t	0.2	-	-	-	0.1	t
1433	*trans*-α-Bergamotene	0.3	t	t	0.1	0.6	0.1	0.1	0.1	0.1	0.1	0.1	0.1	0.1	0.1	0.3	0.1	0.1	0.2	0.2	0.1	0.5	0.1	0.1
1435	Aromadendrene	t	t	t	t	t	-	-	-	t	t	-	-	t	t	t	t	t	0.2	-	-	t	-	t
1443	6,9-Guaiadiene	-	t	-	-	-	-	t	t	t	0.1	-	-	t	t	-	-	t	-	-	t	-	0.1	0.1
1446	*cis*-Muurola-3,5-diene	t	t	-	0.1	t	t	-	-	-	0.1	-	-	-	-	-	-	-	t	t	-	-	-	-
1451	*trans*-Muurola-3,5-diene	-	-	-	-	t	-	-	0.1	-	-	t	-	0.1	0.1	t	0.2	t	-	t	-	t	0.1	0.1
1454	α-Humulene	0.6	0.4	-	0.6	0.5	0.2	0.1	0.3	0.1	0.3	0.2	t	1.0	0.5	0.6	0.8	0.8	t	0.3	0.1	0.9	0.4	0.5
1457	*allo*-Aromadendrene	0.1	0.1	0	0.2	t	t	t	0.1	t	0.1	t	t	0.3	0.1	0.1	0.2	0.2	0.1	0.1	t	0.1	0.1	0.1
1463	*cis*-Cadina-1(6),4-diene	0.1	0.1	-	0.1	t	t	t	0.2	t	0.1	t	-	0.2	0.1	0.1	0.2	0.1	-	t	-	0.1	0.1	0.1
1473	*trans*-Cadina-1(6),4-diene	0.2	0.2	t	0.2	0.1	0.1	0.1	0.2	0.1	0.2	0.1	t	0.4	0.2	0.2	0.5	0.2	0.2	0.1	0.1	0.3	0.3	0.3

1480	γ-Himachalene	-	-	0.1	-	-	-	-	-	t	-	-	0.2	-	t	-	-	-	0.1	t	-	-	-	-
1484	Germacrene D	0.2	0.2	0.1	0.2	0.3	0.1	t	0.1	0.1	0.1	t	0.2	0.4	t	0.2	0.4	0.2	-	0.1	t	0.3	0.1	0.1
1486	δ-Selinene	t	t	-	t	t	t	-	0.1	-	-	t	-	0.1	t	t	0.1	t	-	t	-	0.1	0.1	0.1
1487	β-Selinene	0.2	0.2	-	0.3	0.1	0.1	0.1	0.3	0.1	0.2	0.1	t	0.7	0.3	0.2	0.3	0.2	-	0.1	t	0.3	0.3	0.2
1490	Viridiflorene	-	-	-	-	-	-	0.1	0.2	0.1	0.3	-	-	-	-	-	-	-	-	-	-	-	-	-
1492	*trans*-Muurola-4(14),5-diene	0.1	0.1	-	0.1	t	t	-	-	-	-	-	-	0.3	0.1	0.1	0.3	0.1	-	0.1	t	0.1	0.2	0.2
1498	α-Selinene	0.2	0.2	-	0.3	0.1	0.1	0.1	0.2	0.1	0.1	0.1	-	0.7	0.2	0.2	0.4	0.3	-	0.1	t	0.3	0.3	0.3
1501	α-Muurolene	0.1	0.1	t	0.1	0.1	t	t	0.1	t	0.1	0.1	t	0.3	0.1	0.1	0.3	0.1	t	0.1	t	0.2	0.2	0.2
1508	β-Bisabolene	0.1	-	t	-	0.4	0.1	t	0.1	t	t	0.1	-	-	t	0.1	t	-	t	t	t	0.1	-	-
1514	γ-Cadinene	0.3	0.2	t	0.4	0.1	0.1	0.1	0.3	0.1	0.3	0.1	t	0.7	0.3	0.3	0.7	0.3	0.1	0.1	t	0.4	0.4	0.4
1516	Cubebol	-	0.1	-	t	-	-	-	-	-	-	-	-	0.2	-	0.1	0.1	-	-	-	-	-	0.1	0.1
1520	*trans*-Calamenene	t	-	-	t	-	-	-	t	t	t	-	t	0.1	0.1	0.1	t	t	t	t	t	0.1	0.1	-
1521	δ-Cadinene	0.7	0.5	t	0.7	0.2	0.3	0.2	0.8	0.2	0.6	0.3	0.1	1.7	0.8	0.7	1.6	0.9	0.1	0.4	0.1	1.1	0.9	0.8
1523	Zonarene	t	-	-	t	-	t	t	0.1	t	0.1	t	-	0.1	t	t	t	t	-	t	-	-	0.1	t
1529	Kessane	-	-	0.1	-	-	t	-	-	t	t	t	0.2	-	t	-	-	-	0.5	t	t	-	-	-
1532	*trans*-Cadine-1,4-diene	t	t	-	t	t	t	-	0.1	t	t	t	-	0.1	t	t	0.1	t	-	t	-	t	0.1	0.1
1537	α-Cadinene	t	t	-	t	t	t	-	t	t	t	t	-	0.1	t	t	0.1	t	-	t	-	t	t	t
1539	α-Calacorene	t	t	-	t	-	t	-	0.1	t	t	t	-	t	t	t	t	t	-	t	-	0.1	t	t
1548	Elemicin	-	-	-	-	-	-	-	-	-	-	-	-	-	-	-	-	-	0.2	-	-	-	-	-
1549	α-Elemol	t	t	-	t	-	t	t	0.1	t	0.1	t	-	0.2	0.1	t	0.1	t	-	t	-	-	0.1	0.1
1560	Germacrene B	t	-	-	-	t	t	-	-	-	-	-	-	-	-	t	-	-	-	-	-	t	-	-
1560	β-Calacorene	t	t	-	t	-	-	-	t	-	-	t	-	0.1	t	t	t	t	-	t	-	0.1	-	-
1567	Palustrol	t	t	-	t	-	t	-	t	t	t	t	-	t	t	t	t	-	-	-	-	t	t	t
1577	Caryophyllene oxide	0.8	0.5	-	0.5	0.2	0.3	0.1	0.5	0.1	0.5	0.3	t	1.0	0.5	0.7	0.9	0.6	1.0	0.3	0.2	1.2	0.9	1.0
1578	Spathulenol	t	0.1	-	t	t	t	-	t	t	t	t	-	0.1	t	t	0.1	t	-	t	-	0.1	0.1	0.1
1585	Ethyl isopropyl phthalate	-	-	-	-	-	-	-	-	-	-	-	-	-	-	-	-	-	0.1	-	-	-	-	-
1592	Viridiflorol	-	0.4		t	t	t	0.5	0.8	0.5	2.1	t	-	0.3	0.1	0.1	0.2	0.1		t	0.1	0.1	1.1	0.7
1605	Ledol	t	-	-	-	-	-	-	t	t	t	t	-	t	t	t	t	-	-	-	-	t	t	t
1612	Humulene epoxide II	0.1	0.1		0.1	t	t	t	0.1	t	0.1	t	-	0.2	0.1	0.1	0.1	0.1	t	t	t	0.2	0.3	0.3
1616	1,10-di-*epi*-Cubenol	0.1	-	-	-	-	t	t	0.1	-	0.1	0.1	-	-	-	-	0.1	0.1	-	-	-	0.2	0.1	0.1
1626	1-*epi*-Cubenol	t	t	-	t	t	t	-	0.1	t	0.1	t	-	0.1	t	t	0.1	t	-	t	-	0.1	0.1	0.1
1640	*epi*-α-Cadinol	0.3	0.2	-	0.3	t	0.2	0.1	0.5	0.1	0.5	0.3	-	0.8	0.4	0.4	0.94	0.2	-	0.1	t	0.6	0.8	0.9
1644	δ-Cadinol	t	t	-	-	-	t	-	-	-	-	t	-	t	t	t	t	-	-	-	-	0.1	t	t
1655	α-Cadinol	0.1		-	-	t	-	-	-	t	-	0.1	-	0.2	0.1	0.1	0.2	t	-	-	t	0.2	-	-

1657	α-Eudesmol	-	t	-	0.1	-	t	t	0.1	-	0.1	-	-	-	-	-	-	-	-	t	-	-	0.2	0.2
1793	α-Phellandrene dimer	t	-	t	0.1	t	t	0.1	-	-	-	t	t	0.2	0.1	0.1	0.1	t	0.2	t	t	0.1	0.1	0.1
1946	α-Springene	t	t	-	t	t	t	t	t	t	t	t	-	-	t	t	0.1	0.1	0.1	t	t	0.1	-	-
1950	(3*E*)-Cembrene A	0.1	0.1	t	0.1	t	t	t	0.1	t	0.1	t	t	0.4	0.1	0.1	0.2	0.1	0.2	0.1	t	0.1	0.1	0.1
1977	*p*-Camphorene	-	t	-		t	t	-	-	t	-	-	-	-	t	t	t	0.1	t	t	t	t	-	-
1992	A-Pinacene (= Cembrene C)	t	t	-	t	-	-	-	t	-	t	-	-	0.1	t	t	t	t	-	t	-	t	t	-
1993	Olealdehyde	-	-	-	-	-	-	0.3	-	-	-	-	-	-	-	-	-	-	-	-	-	-	-	-
2012	Verticilla-4(20),7,11-triene	t	t	t	t	t	t	t	0.1	t	t	t	t	0.1	t	t	0.1	t	0.1	t	t	0.1	0.1	t
2057	Ricinelaidic acid lactone	-	-	-	-	-	-	-	-	-	-	t	t	-	-	-	-	-	-	t	0.1	-	-	-
2123	Cembrenol	0.1	0.1		t	t	t	t	0.1	t	0.1	t	t	0.2	0.2	0.1	0.1	t	0.2	t	t	0.3	0.1	0.1
2143	Verticillatriene isomer	-	-	-	0.2	0.4	-	-	0.5	0.2	0.9	-	-	-	-	-	-	-	-	-	-	-	-	-
2145	Serratol	1.0	0.5	t	-	-	0.1	0.3	-	-	-	0.2	0.1	2.1	1.6	1.6	1.7	0.3	1.3	0.2	0.1	1.7	0.7	0.7
2146	Incensole	-	0.1	-	-	-	-	0.1	-	-	-	-	-	-	-	-	-	-	-	-	-	-	0.2	0.1
2149	Incensyl acetate	t	0.2	-	t	t	t	-	t	-	-	-	-	-	0.3	-	t	-	-	t	-	-	-	-
2260	(9*Z*)-Eicosen-1-ol	-	-	-	-	-	-	0.1	-	-	-	-	-	-	-	-	-	-	-	-	-	-	-	-
2264	*iso*-Incensyl acetate	-	t	-	-	-	-	-	-	-	-	-	-	-	t	-	-	-	0.1	-	-	t	-	-
2257	Incensole oxide	-	-	-	-	-	-	-	-	-	-	-	-	-	-	t	-	-	t	-	-	t	-	-
2330	Copalic acid	-	-	-	-	-	-	-	-	-	-	-	-	-	-	t	-	-	-	-	-	-	-	-
2400	Tetracosane	-	-	-	-	-	-	-	-	-	-	-	-	-	-	-	-	-	-	-	-	-	0.5	-
2464	(13*Z*)-Docosen-1-ol	-	-	-	-	-	-	0.3	-	-	-	-	-	-	-	-	-	-	-	-	-	-	-	-
2678	Methyl commate isomer	-	-	-	-	-	-	-	-	-	-	-	-	-	-	-	-	7.1	-	-	-	-	-	-
2792	Methyl commate B	-	0.9	-	0.1	-	-	-	-	-	-	-	0.3	-	-	-	-	-	-	-	-	-	-	-
2793	24-*nor*-Ursa-3,12-dien-11-one	-	-	-	-	-	-	-	-	-	-	-	-	-	-	-	-	-	-	0.7	-	-	-	-

"-" indicates not detected and "t" indicate trace (≤0.05%).
